# Safety and Efficacy of Minimally Invasive Sacrospinous Ligament Fixation for Apical Pelvic Organ Prolapse in Older Women

**DOI:** 10.3390/jcm13185520

**Published:** 2024-09-18

**Authors:** Ronen S. Gold, Jonatan Neuman, Yoav Baruch, Menahem Neuman, Asnat Groutz

**Affiliations:** 1 Urogynecology Unit, Department of Obstetrics and Gynecology, Lis Maternity and Women’s Hospital, Tel-Aviv Medical Center, Medical School, Tel Aviv University, Tel-Aviv 6423906, Israel; 2 Medical School, Semmelweis University, 1085 Budapest, Hungary; 3 The Urogynecology Service, Assuta Medical Centers, Medical School, Ben Gurion University, Beer-Sheva 8410501, Israel

**Keywords:** sacrospinous ligament fixation, pelvic organ prolapse, surgery, EnPlace^®^, older women

## Abstract

**Background:** This study aimed to evaluate the safety and efficacy of minimally invasive sacrospinous ligament (SSL) fixation of apical pelvic organ prolapse (POP) in older patients compared to younger patients. **Methods:** A cohort of 271 older (≥65 years) patients (mean age 71.8 ± 5.2 years) and 60 younger patients (mean age 47.6 ± 7.1 years) with stage III or IV apical POP who underwent SSL fixation by the EnPlace^®^ device was retrospectively analyzed. The age range of older patients was further divided into early old (65–74 y, N = 209), old (75–84 y, N = 58), and late old (>85 y, N = 4). Patient characteristics, surgical safety, and 6-month postoperative outcomes were compared between the four age groups. **Results:** Duration of surgery and blood loss were similar among all age groups. Most patients (99.4%) were discharged on the day of surgery or the day after. Subjective patient satisfaction rates were high among all patients. Point C measurements at six months postoperatively were less favorable among the younger patients. Furthermore, four (6.7%) younger patients versus six (2.2%) older patients required surgical repair of recurrent apical POP within the follow-up period. **Conclusions:** The short-term outcomes of minimally invasive SSL fixation suggest that it is a safe and effective procedure for significant apical POP repair among older patients.

## 1. Introduction

Pelvic organ prolapse (POP) is very common among women, with up to 11% lifetime risk for surgical repair [1,2,3]. The etiology of POP is multifactorial, but the two main risk factors are vaginal deliveries and aging. As much as half of women with POP are asymptomatic, and for these women, no therapeutic intervention is required. Significant POP is usually associated with a variety of lower urinary tract symptoms (LUTS), such as vaginal bulging or discomfort, urinary incontinence, voiding dysfunction, and recurrent urinary tract infections (UTIs). Additionally, women with significant POP may present with vaginal bleeding, sexual dysfunction, and/or colorectal symptoms. In cases of neglected significant POP, chronic bladder outlet obstruction may cause irreversible hydronephrosis and renal failure. There are several classification methods to define the severity of POP. Most clinicians, regardless of the specific classification in use, refer to a combination of a pelvic organ bulging beyond the vaginal hymen and bothersome POP-related symptoms as clinically significant POP.

Therapeutic alternatives for significant POP include vaginal pessaries or reconstructive pelvic surgery. There are different types of vaginal pessaries to provide a mechanical support for POP. The success of conservative management with pessaries varies depending on the specific clinical protocol and patient characteristics. However, regardless of the protocol, a vaginal pessary is a foreign body inside the vagina and may be associated with infections, erosion, or expulsion. Additionally, vaginal pessaries are not ideal for sexually active women and require periodic maintenance [4]. Women with significant symptomatic POP who are unable or unwilling to use a pessary should be offered reconstructive pelvic surgery as an alternative treatment option.

The number of older women is projected to triple by 2050 [5], leading to a significant increase in older patients undergoing POP surgery. When choosing the surgical approach for older patients, it is important to consider this population’s specific characteristics and needs. Studies have indicated a higher rate of serious complications following POP surgery in older patients, with severe complications occurring in 9.0% to 25% of the very old population [6]. These complications include major cardiac, neurologic, and hematologic morbidities, as well as an increased risk of mortality. Various risk factors, such as the duration of surgery, coronary artery disease, and peripheral vascular disease, have been linked to these specific complications [7]. Additionally, major surgery might lead to postoperative delirium, which is characterized by a sudden change in mental status. In a recently published study, 165 women aged ≥60 years (mean 72.5 ± 6.1 years) who underwent POP surgery were screened for postoperative delirium on the day of hospital discharge and at 1, 3, 5, and 7 days thereafter [8]. The incidence of positive screening for postoperative delirium was 12.1%. Notably, patients with postoperative delirium at any time after surgery were significantly older. Therefore, understanding the safety and effectiveness of POP surgery in older patients is essential for optimizing patient safety and outcome results. Yet, there is a lack of data on the safety and efficacy of POP surgery among older patients [9,10,11].

**Figure 1:**
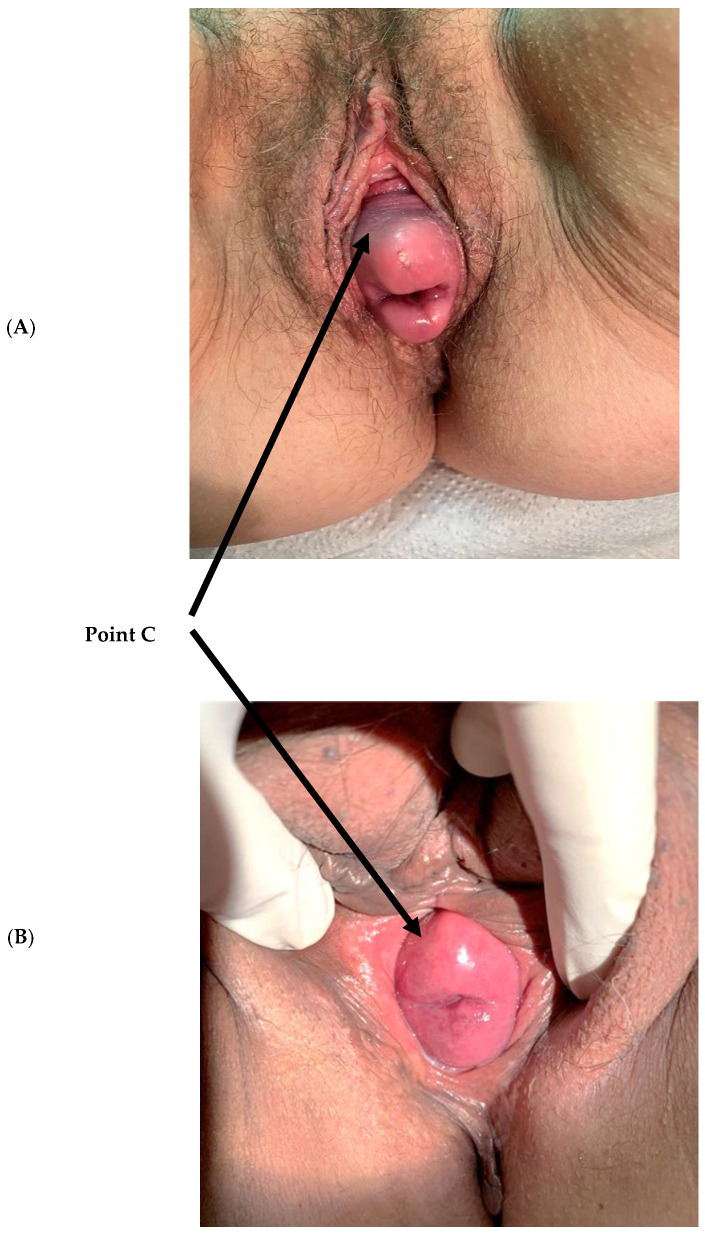
Apical pelvic organ prolapse. (**A**) Point C at + 2.5 cm; (**B**) point C at +0.5 cm.

Apical POP is defined as prolapse of the uterus, uterine cervix, or vaginal vault (Figure 1). Apical prolapse is the dominant component in about 20% of patients with POP; however, in most cases, there is a concomitant prolapse of either the anterior (cystocele) and/or the posterior (rectocele) compartments, and in some patients, there is also concomitant stress urinary incontinence (SUI). Significant apical POP is characterized by the bulging of the prolapse beyond the hymen, accompanied by various pelvic floor symptoms. Surgical repair of significant apical POP is challenging. There are different approaches to apical repair: transabdominal, transvaginal, with or without uterine preservation, and with or without the use of mesh [11,12]. The transvaginal approach is associated with low rates of complications and a relatively quick recovery [10]. However, following the FDA warnings regarding vaginal mesh for POP surgery [13], the use of vaginal mesh has been mostly abandoned and is much less utilized for POP repair [14]. The transabdominal approach is more popular due to its long-term durability and low recurrence rates. Thus, laparoscopic or robotic transabdominal sacrocolpopexy has become the reference standard for apical POP repair [12,14,15]. Yet, the transabdominal approach is associated with higher complication rates and requires general anesthesia, which may further increase postoperative morbidity [10].

**Figure 2:**
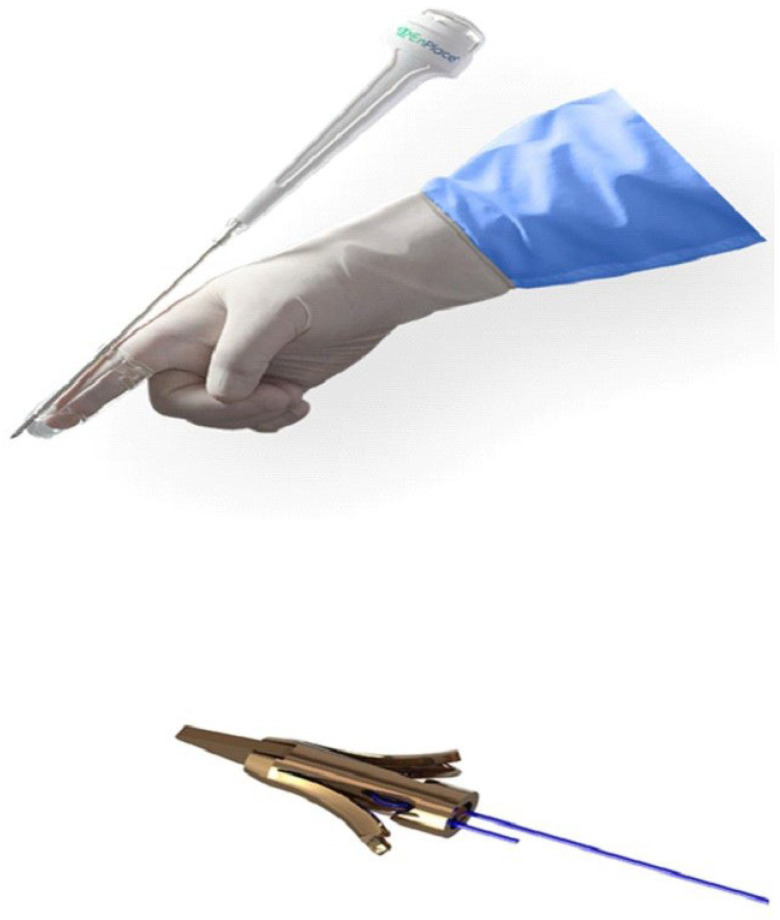
The EnPlace^®^ device (with permission FEMSelect).

Sacrospinous ligament (SSL) fixation is a well-established transvaginal surgical approach to correct apical POP. It can be performed either unilaterally or bilaterally; however, the technique requires deep dissection to explore the SSL and advanced transvaginal surgical skills. The EnPlace^®^ device is a minimally invasive approach for transvaginal SSL fixation of apical POP (Figure 2). The device enables SSL fixation without extensive dissection or mesh. It has been proven safe and effective [16,17]. The present study aimed to evaluate the short-term safety and efficacy of transvaginal SSL fixation by the EnPlace^®^ device for stage III or IV apical POP in older patients compared to younger patients.

## 2. Methods

Old age was defined as 65 years and older. A cohort study of 331 consecutive older and younger patients who underwent transvaginal EnPlace^®^ SSL fixation in two university-affiliated medical centers was retrospectively analyzed from January 2019 to December 2023. All patients underwent surgical repair by using the EnPlace^®^ device for significant (stage III–IV) apical POP. The Institutional Review Board approved the study protocol (Tel Aviv Sourasky Medical Center 22-0636-TLV). Demographic, clinical, intra-operative, and post-operative data were retrieved from a computerized database. Preoperative data included age (years), parity, severe comorbidities such as ischemic heart diseases, neurologic impairment, metabolic disorders, frailty, prior hysterectomies, either transvaginal or transabdominal, LUTS, and severity of POP. POP severity was assessed using the POP-Quantification (POP-Q) measurements [11]. This method is widely used for classifying the type and severity of POP and is recommended by all professional societies and organizations. Conceptually, it comprises six vaginal locations with regard to the hymen and three anatomical lengths [18,19]. Point C, the lowest part of the uterine cervix or the vaginal vault, represents apical prolapse. POP severity is further categorized into stages I–IV. In stage III, the most distal portion of the prolapse extends more than 1 cm distal to the hymen but no further than 2 cm less than the total vaginal length (TVL). In stage IV, the most distal portion of the prolapse protrudes to greater than TVL-2 cm, i.e., complete to nearly complete apical eversion. Significant POP was defined as subjective prolapse-related symptoms and objective stages III–IV prolapse by the POP-Q classification.

The EnPlace^®^ device (FEMSelect, Tel Aviv, Israel) enables minimally invasive transvaginal fixation of the uterine cervix, or vaginal vault, into the SSL. The device consists of a sharp Nitinol anchor deployed in a transvaginal applicator and attached to a surgical suture. This delivery system allows for precise piercing through vaginal walls into the SSL. Once the anchor is fixed into the SSL, the attached surgical suture is sewn into the vaginal apex, either at the uterine cervix or vaginal vault. The technique does not require extensive vaginal dissection and can be performed under regional anesthesia. Exclusion criteria included genital anomalies, previous pelvic radiation therapy or inflammatory disease, malignancy, or a known allergy to nickel or nitinol.

The study cohort was divided into two groups: 271 older patients (mean age 71.87 ± 5.23 years; range 65–92 years) and 60 younger patients (mean age 47.6 ± 7.1 years; range 36–64 years). The older group was further divided into three subgroups: early old (65–74 y, N = 209), old (75–84 y, N = 58), and late old (≥85 y, N = 4). All 331 patients underwent transvaginal SSL fixation of stage III–IV apical POP by using the EnPlace^®^ device. A native-tissue repair was performed for patients with either anterior (cystocele) and/or posterior (rectocele) POP. This involved anterior and/or posterior colporrhaphy. The anterior colporrhaphy included double-layer sutures of the pubocervical fascia, while the posterior colporrhaphy involved repair of the rectovaginal fascia and perineoplasty. Patients with SUI, or occult SUI per preoperative urodynamic evaluation, underwent a concomitant inside-out trans-obturator mid-urethral sling (Gynecare TVT Obturator System; Ethicon Inc., Somerville, NJ, USA).

Intraoperative data included the duration of surgery (minutes), estimated blood loss (ml), surgical complications, and measurements of point C by POP-Q classification at the end of surgery. Follow-up assessment was carried out at six weeks, three months, and six months postoperatively. The postoperative evaluation included anatomical and functional cure rates, pain, dyspareunia, LUTS, and UTIs. Anatomical cure was defined as no apical prolapse beyond stage II by the POP-Q classification and no significant prolapse-related pelvic floor symptoms. Patient-reported outcomes were evaluated by decision regret or satisfaction on a scale from 0 to 100 according to the Satisfaction with Decision Scale-Pelvic Floor Disorders [20]. Surgical safety and efficacy were compared between all age groups.

Statistical analysis was performed using the Student *t*-test for continuous data or Fisher’s exact test for categorical data. Data are summarized as mean± standard deviation (SD), or percentage, according to the variables. Statistical tests were two-sided; a *p*-value < 0.05 was considered statistically significant. SPSS software version 27 (IBM Corporation, Armonk, NY, USA) was used for the statistical analysis.

## 3. Results

Data from 209 early-old (mean age 69.5 ± 2.7 years), 58 old (mean age 79.1 ± 2.6 years), and four late-old (mean age 89.2 ± 2.5 years) patients were analyzed and compared to 60 younger patients (mean age 47.6 ± 7.1 years). Patient characteristics of the older subgroups versus younger patients are presented in Table 1. As expected, a direct correlation was demonstrated between the patient’s age and preoperative comorbidities. Overall, preoperatively, 205 (61.9%) patients had stage III or IV uterine prolapse, and 126 (38.1%) had stage III or IV vaginal vault prolapse. However, stage III or IV vaginal vault prolapse was significantly more common among older patients (34.3% versus 16.7%; *p* < 0.05). The preoperative severity of the apical prolapse was similar among all age groups, with similar preoperative mean point C measurements.

Intraoperative data of the older subgroups versus younger patients are presented in Table 2. Overall, the mean duration of the surgical procedure and mean estimated intraoperative blood loss were similar among older and younger patients (23.9 ± 6.1 min versus 32.8 ± 7.3 min and 24.4 ± 6.9 mL versus 30.4 ± 13.9 mL, respectively).

Point C measurements by POP-Q classification before surgery, at six weeks, and six months postoperatively are presented in Table 3. Measurements at six months postoperatively were statistically less favorable among the younger patients (−3.7 ± 0.9 versus −5.4 ± 0.9, respectively; *p* < 0.05). 

There were three early postoperative complications, all among early old patients: two cases of retroperitoneal hematoma that were managed conservatively and one case of intractable pain that required removal of fixation suture. Most patients (99.4%) in all age groups were discharged on the day of surgery or the day after. Seven (2.6%) older patients experienced early postoperative obstructed defecation. These patients were managed successfully with stool softeners. There were no significant postoperative de novo LUTS among all age groups. Patient-reported outcomes were evaluated by decision regret or satisfaction on a scale from 0 to 100. Mean satisfaction scores were high (>90) among all age groups. 

Within the follow-up period, four (6.7%) of the 60 younger patients required a recurrent surgical repair of apical POP, only one (25%) of whom had preoperative vaginal vault prolapse. In addition, two other young patients (3.33%) with recurrent apical POP chose to be treated with a vaginal pessary. Of the 271 older patients, 6 (2.2%) required a recurrent surgical repair of apical POP, two (33.33%) of whom had preoperative vaginal vault prolapse. Additionally, 16 (5.9%) other older patients developed a recurrent stage II asymptomatic apical POP, which did not require any intervention, neither conservative nor surgical.

## 4. Discussion

Pelvic reconstructive surgery poses unique challenges for older patients in terms of selecting the appropriate surgical approach and managing perioperative age-related complications. The elderly population is characterized by co-morbidities, some of them significant, including mobility disorders, cognitive impairment, and frailty. These characteristics are especially challenging when it comes to pelvic floor reconstruction surgeries, which are mainly intended to improve quality of life. In addition to significant co-morbidities and frailty, any surgical intervention may cause postoperative delirium in older patients. Delirium is a sudden neuropsychiatric syndrome associated with adverse consequences. A recent systemic review and meta-analysis analyzed the results of 49 studies with a total of 26,865 patients screened for delirium after non-cardiac surgeries [21]. The overall rate of postoperative delirium was found to be 23.8%. Type of anesthesia and preoperative cognitive status were identified as significant risk factors for postoperative delirium, with an increased rate of up to 28% after general anesthesia. Similarly, Ackenbom et al. reported a 12.1% postoperative delirium in older patients undergoing POP surgery [8]. Thus, minimally invasive procedures, which require a shorter duration of surgery and do not require general anesthesia, are preferable among older patients [7,8,10,11]. The definition of older age varies among populations. An older person is defined by the United Nations and World Health Organization as a person who is over 60 years of age [22,23]. However, most Western countries use the cutoff of 65 years to define older age [24]. The modern era is characterized by the development of medical technologies that enable better health and longer lifetimes than in the past. Since the world population is aging, the older age group cannot be generalized as a homogeneous group, and it is necessary to define different age subgroups accurately, especially for medical and surgical purposes. Previous studies have suggested dividing the older age group into youngest-old (ages 65–74 years), middle-old (ages 75–84 years), and oldest-old (≥85 years) age subgroups [25,26]. This sub-classification enables a better understanding of the surgical challenges faced by different age groups. In the present study, the safety and efficacy of apical POP repair were evaluated according to these subcategories of the older age groups. To the best of our knowledge, previously published studies regarding POP surgery did not address the specific clinical characteristics of the various subcategories of old age. Additionally, the study assessed the outcome results of minimally invasive SSL fixation, which enables shorter surgery under regional anesthesia.

The safety and efficacy of POP surgery in older patients have only been investigated in a small number of studies. Farthmann et al. investigated a series of 91 older patients (mean age 84.4 ± 3.1 years, range 80–92 years) who underwent POP surgery [27]. The mean duration of surgery was 81 ± 45 min; 94.5% of patients were under general anesthesia. Postoperatively, six patients (6.6%) required intensive care monitoring. The mean length of hospital stay was eight days. Chapman et al. analyzed perioperative data of 20,567 patients aged 65–79 years, 3088 patients aged ≥80 years, and 27,403 younger patients aged 45–64 years who underwent surgical repair of POP [6]. The rate of severe complications among the younger patients was 4.5%, compared with 4.7% among old patients (odds ratio (OR 1.0, 95% CI 0.9–1.1) and 9.0% among very old patients (OR 2.1, 95% CI 1.8–2.4). Specifically, very old patients had significant risks of cardiac complications (OR 11.9, 95% CI 6.2–23.0), stroke (OR 26.6, 95% CI 5.4–131.8), and mortality (OR 39.9, 95% CI 8.6–184.7). On multivariate logistic regression analysis, the very old age was independently associated with severe complications (adjusted OR 2.01, 95% CI 1.8–2.3). The authors concluded that POP surgery is associated with a significant risk of severe perioperative morbidity among patients older than 80 years, independent of frailty and medical or surgical risk factors. Joukhadar et al. analyzed the 3–6 month outcome results of 407 patients who underwent POP and/or SUI surgery [28]. Of the 407 patients, 129 were 70 years or older. The results showed that more younger patients underwent laparoscopic sacropexy, whereas more older patients underwent mesh-assisted vaginal POP repair. Surgical success rates were high in both age groups (84.8–93.5%). The investigators concluded that “*both prolapse and SUI can be treated surgically in 70-year-old or older patients with good results, and these patients should be offered the same range of surgical options as their younger counterparts*”. These conflicting results prompt the need for further investigation of the safety and efficacy of POP surgery among older patients.

Less is known regarding the safety and efficacy of apical POP repair among old patients. Chill et al. retrospectively studied a cohort of 271 patients who underwent apical POP repair by uterosacral ligament suspension [29]. Of the 271 patients, 209 (77%) were younger than 70 years, and 62 (23%) were 70 years or older. Most patients in both age groups underwent transvaginal uterosacral ligament suspension combined with anterior and/or posterior colporrhaphies. Half of the patients in both age groups also underwent concomitant mid-urethral sling. Mean follow-up was 19 ± 20 and 17 ± 16 months for the young and older age groups, respectively. Anatomical and composite outcome success rates were higher among the younger patients (76% vs. 56%, *p* <  0.01; and 70% vs. 54%, *p* =  0.02, respectively); however, a multivariant analysis failed to reveal statistically significant differences between the age groups. The investigators concluded that patients over the age of 70 undergoing uterosacral ligament suspension for apical POP have comparable outcomes to younger patients. Recently, Barba et al. retrospectively analyzed 65 patients, with a median age of 81.3 years, who underwent transvaginal hysterectomy combined with an apical suspension procedure for stage II–IV symptomatic POP [30]. The study assessed both anatomical and functional outcomes. Subjective postoperative satisfaction was evaluated using the Patient Global Impression of Improvement (PGI-I) score. Almost all patients (96.9%) underwent a concomitant anterior and/or posterior native tissue repair. During surgery, eight patients experienced blood loss of 500 mL or more, with no other intraoperative complications recorded. The median follow-up duration was 23 ± 20 months. There were no significant perioperative or postoperative complications. Postoperative anatomical recurrence of POP was observed in 10.7% of the patients (7.69% in the anterior compartment, 1.5% in the apical compartment, and 4.6% in the posterior compartment). Notably, none of these POP recurrences necessitated further surgical intervention. All patients reported satisfaction with the surgery, with a median PGI-I score of 1.12, indicating very high subjective cure rates. Furthermore, objective cure rates were 89.5%. Overall, the study supports the utilization of transvaginal hysterectomy combined with apical suspension, particularly employing high uterosacral ligament suspension, as a safe and effective primary surgical approach with favorable outcomes, even in elderly patients.

The present study aimed to evaluate the short-term safety and efficacy of minimally invasive transvaginal SSL fixation by the EnPlace^®^ device for stage III–IV apical POP in 271 older patients compared to 60 younger patients. The older age group was further divided into three subgroups: early old (65–74 y, N = 209), old (75–84 y, N = 58), and late old (≥85 y, N = 4). Preoperatively, older patients had higher rates of significant comorbidities. Duration of surgery and blood loss were similar among the young and older age groups (23.9 ± 6.1 min versus 32.8 ± 7.3 min and 24.4 ± 6.9 mL versus 30.4 ± 13.9 mL, respectively). Further, there were no statistically significant differences between the specific age categories with regard to these measurements. Most patients (99.4%) were discharged on the day of surgery or the day after. There were three cases of early postoperative complications among the early old patients: two cases of retroperitoneal hematoma that were managed conservatively and one case of pelvic pain that required cutting and releasing of the fixation suture. At six months follow-up, subjective patient satisfaction rates were high among all age groups. However, point C measurements at six months postoperatively were less favorable among the younger patients. Furthermore, four younger patients (6.7%) versus six older patients (2.2%) required a recurrent apical POP repair within the follow-up period. It is possible that this finding is due to more vigorous physical activity among young patients compared to older patients.

The study’s strengths include a large number of patients, a sub-analysis of older age groups, strict postoperative follow-up, and the absence of patients lost to follow-up. The study has several drawbacks. Firstly, the outcome measures, particularly regarding surgical safety in older patients, are inherently biased, as only patients without severe comorbidities are candidates for surgery. Furthermore, the participants constituted a homogeneous group of patients operated on by trained surgeons. Thus, results may differ in other populations and with less competent surgeons.

As older patients are at a higher risk of having POP surgery, there is a need for an operation that is minimally invasive, short, can be performed under regional anesthesia, and has fewer complications with high success rates. Additionally, a transvaginal approach is more economical than a laparoscopic or robotic transabdominal approach [17]. The transvaginal EnPlace^®^ SSL fixation does not require mesh or deep dissection. Therefore, the operative time is short, and the complication rate is low. Additional studies are required to enable personalized medicine, optimize surgical results, and improve the quality and safety of surgery in older patients.

## 5. Conclusions

The present study aimed to evaluate the short-term safety and efficacy of minimally invasive transvaginal SSL fixation by the EnPlace^®^ device for stage III–IV apical POP in 271 older patients compared to 60 younger patients. The older age group was further divided into three subgroups: early old (65–74 y, N = 209), old (75–84 y, N = 58), and late old (>85 y, N = 4). The results of the present study suggest that transvaginal minimally invasive SSL fixation is a safe and effective procedure to correct significant apical POP in older patients. The study’s main strengths include the large cohort as well as the sub-analysis of the older age groups. However, the present cohort consists of a relatively homogeneous group of patients operated on by trained surgeons. Thus, results may differ in other populations and with less competent surgeons. Further research is required on different surgical techniques for POP repair in older patients.

## Figures and Tables

**Table 1:** Patient characteristics by age groups.

Mean ± SD, or N (%)	Early Old 65–74 Years 209 (77.12%)	Old 75–84 Years 58 (21.4%)	Late Old ≥85 Years 4 (1.48%)	Young <65 Years 60
Age (y)	69.5 ± 2.7	79.1 ± 2.6	89.2 ± 2.5	47.6 ± 7.1
Parity	3.1 ± 1.4	3.5 ± 1.7	3 ± 0.8	3.3 ± 2.1
Severe comorbidities *	0.9 ± 0.8	1.2 ± 0.8	3 ± 1.4	0.3 ± 0.7 **
Prior hysterectomy	69 (33%)	24 (41.4%)	0	10 (16.7%)
Preoperative point C (cm)	+1.45 ± 1.98	+2.81 ± 1.7	+1.25 ± 0.5	+1.4 ± 2.2

* Severe comorbidities: severe hypertension, diabetes, chronic obstructive pulmonary disease, ischemic heart disease, congestive heart disease. ** *p* < 0.05.

**Table 2:** Intra-operative data by age groups.

Mean ± SD, or N (%)	Early Old 65–74 Years 209 (77.12%)	Old 75–84 Years 58 (21.4%)	Late Old ≥85 Years 4 (1.48%)	Young <65 Years 60
Concomitant colporrhaphies	204 (97.6%)	54 (93.1%)	4 (100%)	58 (96.7%)
Concomitant Mid Urethral Sling	43 (20.6%)	4 (6.9%)	2 (50%)	9 (15%)
Duration (min)	24.2 ± 6.3	21.9 ± 4.2	27.6 ± 6.5	32.8 ± 7.3
Bleeding (cc)	24.8 ± 7.1	22.4 ± 5	31.3 ± 10.3	30.4 ± 13.9

**Table 3:** Point C measurements by POP-Q classification.

Mean ± SD, or Number (%)	Early Old 65–74 Years 209 (77.12%)	Old 75–84 Years 58 (21.4%)	Late Old ≥85 Years 4 (1.48%)	Young <65 Years 60
Point C (cm):				
Preoperative	+1.45 ± 1.98	+2.81 ± 1.7	+1.25 ± 0.5	+1.4 ± 2.2
Six weeks postoperative	−5.6 ± 0.5	−5.5 ± 0.5	−5.5 ± 0.6	−5.4 ± 0.95
Six months postoperative	−5.4 ± 0.95	−5.4 ± 0.9	−5.25 ± 0.9	−3.7±0.9 *

* *p* < 0.05.

## Data Availability

The original contributions presented in the study are included in the article; further inquiries can be directed to the corresponding author/s.
